# Risk of Ischemic Heart Disease Associated with Primary Dysmenorrhea: A Population-Based Retrospective Cohort Study

**DOI:** 10.3390/jpm12101610

**Published:** 2022-09-29

**Authors:** Chung-Hsin Yeh, Chih-Hsin Muo, Fung-Chang Sung, Pao-Sheng Yen

**Affiliations:** 1 Department of Nursing, College of Nursing and Health, Da-Yeh University, Changhua 51500, Taiwan; 2 Department of Neurology, Yuan Rung Hospital, Changhua 51045, Taiwan; 3 Management Office for Health Data, China Medical University Hospital, Taichung 40447, Taiwan; 4 Department of Health Services Administration, China Medical University College of Public Health, Taichung 40447, Taiwan; 5 Department of Food Nutrition and Health Biotechnology, Asia University, Taichung 41354, Taiwan; 6 Department of Neuroradiology, Kuang Tien General Hospital, Taichung 43303, Taiwan

**Keywords:** claims data, dysmenorrhea, ischemic heart disease, nested case-control study, retrospective cohort study

## Abstract

The awareness on ischemic heart disease (IHD) in women with dysmenorrhea is insufficient. We utilized the National Health Insurance Research Database (NHIRD) of Taiwan to evaluate this relationship. From the claims data, we established a cohort of women aged 15–50 years with primary dysmenorrhea diagnosed from 2000 to 2008 (*n* = 18,455) and a comparison cohort (*n* = 36,910) without dysmenorrhea, frequency matched by age and diagnosis date. Both cohorts were followed until the end of 2013 to assess IHD events. With 75% of study population aged 15–29 years, the incidence of IHD was greater in the dysmenorrheal cohort than in the comparison cohort (1.93 versus 1.18 per 10,000 person-years), with an adjusted hazard ratio of 1.60 (95% confidence interval [CI] = 1.38–1.85). The incidence increased with age and the rate of increase was greater in the dysmenorrheal cohort than the comparison cohort. Nested case-control analysis in the dysmenorrhea cohort showed that IHD risk was also associated with hypertension and arrhythmia, with adjusted odds ratios of 2.50 (95% CI = 1.64–3.81) and 3.30 (95% CI = 2.25–4.86), respectively. Women with dysmenorrhea are at a higher risk of developing IHD, particularly for older patients and patients with comorbidity.

## 1. Introduction

Cardiovascular disease (CVD) is the leading cause of death worldwide, with an especially high prevalence in wealthier countries [1,2]. The Global Burden of Diseases, Injuries, and Risk Factors Study (GBD) 2019 of the World Health Organization estimated the prevalence of CVD increased from 271 million to 523 million during the recent 20-year interval in 204 countries and territories, while death numbers from CVD surged 50% during the same period [3,4].

Ischemic heart disease (IHD), also called coronary heart disease (CHD), is a heart disease caused by the stenosis of coronary arteries, leading to an insufficient blood supply to the cardiac muscle. IHD is the most common cause of CVD deaths [2,5], and there were 197 million cases globally in 2019. Among those, 182 million became disabled and 9.14 million died from the disease [6]. Hypertension, hyperlipidemia, diabetes, obesity, smoking, and drinking are known risk factors for IHD, affecting both men and women [6,7]. However, women have a higher mortality rate after acute coronary syndrome (ACS) than their male counterparts, including younger women. Women of <45 years old have been reported at a higher risk than men of same age with an odds ratio (OR) of 1.30 (95% CI = 1.23–1.36) [8]. A study in Germany found the age-adjusted in-hospital mortality from ACS was 1.5-fold higher in women than in men (87 versus 57 per 1000 person-years) [9]. As early as the last century, some investigators found the mortality from myocardial infarction during hospitalization was 2-fold higher in women than in men among patients less than 50 years old [10]. Younger women of <55 years old are also more likely to be diagnosed with acute coronary syndrome and unstable angina than older women [11,12].

Since mortality risk from IHD between younger women and younger men is greater than that between older women and older men [13,14], we suspect the age-related difference of IHD risk among women is associated with estrogen levels [15,16]. Women experience menstrual cycles with rising and falling levels of estrogen, testosterone, and progesterone in a fluctuating pattern monthly. The first menstruation starts at menarche and ends with menopause [17]. Women may experience dysmenorrhea, a menstruation with severe pain and cramps in the lower abdomen. Symptoms include sweating, headache, nausea, vomiting, diarrhea, and fear and anxiety around the menstruation period about the appearance of dysmenorrhea symptoms. Primary dysmenorrhea (PD) presents as painful menstruation without known pelvic organic disease and is more prevalent in younger women. Secondary dysmenorrhea (SD) is caused by other pelvic disorders [18,19].

The prevalence rate of dysmenorrhea varies among populations. An earlier review reported the prevalence of dysmenorrhea varying between 67% and 90% in women 17–24 years old [20]. The high prevalence of PD was also found in female students [21,22]. The MDOT study revealed an up to 93% prevalence rate of PD in Australian senior high school girls [21], while over 80% universities girl students were reported with PD in Lebanon [22]. In Taiwan, reported prevalence rates varied from 73.3% to 90.7% among women aged 16–40 years [23,24,25]. Most of the students were self-medicated, only about 30–40% of victims sought healthcare institutes [21,22], even less in some populations [26]. Dysmenorrhea significantly affects quality of life, disturbing school performance and work efficiency and leading to many adolescent and young women being absent from school and work due to the pain [19,27].

The normal menstrual cycle is regulated by the complex secretion and interaction of ovarian hormones along the hypothalamus-pituitary-gonadal axis. The development of PD is associated with the dysregulation of female hormones, including estrogen, follicle-stimulating hormone (FSH), luteinizing hormone (LH), 17β-estradiol, and others [28]. Studies have shown that PD, hypertension, and various kinds of cardiac disease are associated with Estrogen receptor 1 gene polymorphisms [29] and increased prostaglandin-related inflammatory biomarkers production [30,31]. These reports have begun to elucidate the potential risks and mechanisms of developing cardiac disorders, including hypertension, and IHD, in women with PD. Since this hormonal dysregulation increases the risk of IHD [15,16], we utilized claims data of patient cases in the National Health Insurance Research Database (NHIRD) of Taiwan to investigate the relationship between PD and the risk of developing IHD.

## 2. Materials and Methods

### 2.1. Data Source

This retrospective cohort study used the Taiwan Longitudinal Health Insurance Database (LHID) obtained from National Health Insurance Administration, Ministry of Health and Welfare of Taiwan. The database included medical claims of one million insured people randomly selected from 23 million residents who were enrolled in the insurance program in 2000. There were no significant differences in distributions of sex, birth-year and income between people in LHID 2000 and in all insured residents. The database consisted of demographic status of enrollees and claims for outpatient and inpatient care, with information on treatments, medications, and cost available from 1996 to 2013. In LHID, diseases were coded with International Classification of Diseases, Ninth Revision, Clinical Modification (ICD-9-CM), and medicines were prescribed using the anatomical therapeutic chemical (ATC) classification system [32]. This study was approved by the Research Ethics Committee at China Medical University and Hospital, Taiwan (CMUH104-REC2-115). Insurance identification numbers were replaced with surrogate numbers to protect patient privacy.

### 2.2. Study Cohorts

**Figure 1:**
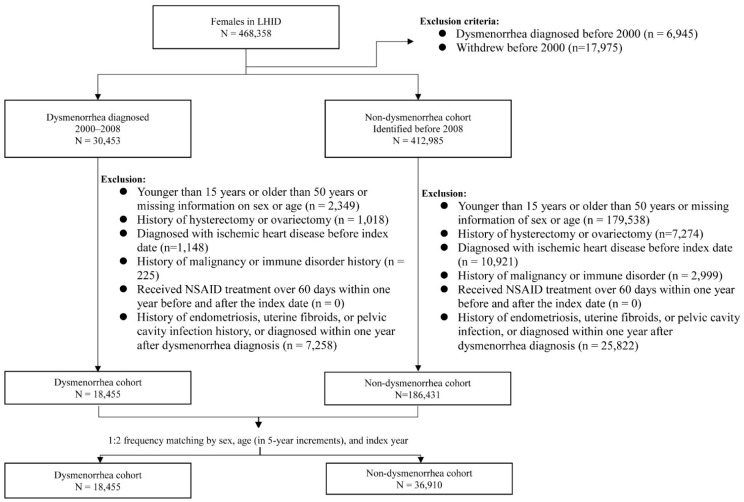
Flow chart for establishing study cohorts.

Women with newly diagnosed dysmenorrhea (ICD-9-CM 625.3) from 2000 to 2008 were identified as the potential dysmenorrhea cohort. We excluded those aged < 15 years old, >50 years old, or those with missing information on age and gender. We also excluded those with the history of pelvic disorders including endometriosis, uterine fibroids, pelvic cavity infection, hysterectomy and ovariectomy, and ischemic heart disease, malignancy, or immune disorder at baseline (Figure 1). Women who received nonsteroidal anti-inflammatory drug (NSAID) treatment for more than 60 days within one year before and after the dysmenorrhea diagnosis were also excluded. The date of dysmenorrhea diagnosis was defined as the index date. The comparison cohort was selected from women without a history of dysmenorrhea, applying the same exclusion criteria used for selecting the dysmenorrhea cohort. For each dysmenorrhea subject, two comparison subjects were selected in the comparison cohort, frequency matched by age and index-year. The method of study cohort selection in this study provided a dysmenorrhea cohort of young women with PD without SD. The potential confounding effects of ischemic heart disease, malignancy, immune disorder and long NSAID uses were also reduced for both cohorts.

### 2.3. Baseline Comorbidity, Medicine Use, and Outcome

We considered baseline comorbidities of diabetes (ICD-9-CM250), hypertension (401–405), hyperlipidemia (272.0–272.4), arrhythmia (427), thyroid disease (240–246), and migraine (346) potential covariates associated with the development of IHD [2,33,34].

NSAID (ATC code: M01A, and M02A) and female hormones (ATC: G03) used within one year before or after the index date were also considered covariates. All study subjects were followed from the index date until the diagnosis of IHD (ICD-9-CM 410-414), or until they withdrew from the LHID, or the end of 2013, whichever came earlier.

### 2.4. Statistical Analysis

Baseline distributions of age (15–29, 30–39, and 40–50 years), comorbidities, and medications were compared between the dysmenorrhea and comparison cohorts. The Chi-square test was used to test the categorical variables, and the *t*-test was used to examine the difference between the mean ages of the two cohorts. Cumulative incidences of IHD were estimated and plotted using Kaplan-Meier analysis, and the difference between two cohorts was tested using the log-rank test. The incidence rate (IR) of dysmenorrhea was estimated using the sum of incident IHD cases divided by the sum of follow-up years (person-years) for each cohort. Cox proportional hazards regression was used to estimate the hazard ratio and 95% confidence interval (CI) for IHD in the dysmenorrhea cohort compared to the non-dysmenorrhea cohort. Significant variables identified in the Cox regression were included in multivariable Cox model to estimate the adjusted hazard ratio (aHR). The interactions between dysmenorrhea and covariates were also examined. We further performed a nested case-control analysis to identify factors associated with developing IHD for the dysmenorrhea cohort. We used logistic regression to examine the odds ratios (OR) of IHD associated with covariates. Multivariable logistical regression was used to estimate the adjusted odds ratio (aOR). We used SAS software Version 9.4 (SAS Institute Inc., Cary, NC, USA) to perform data analyses with a statistical significance level set at *p* < 0.05.

## 3. Results

The study population consisted of 36,910 age- and index-year-matched comparison women and 18,455 women with PD in this study (Table 1). The mean age was close to 26 years, and 75% of women were aged < 30 years. Compared with the non-dysmenorrhea cohort, the dysmenorrhea cohort had a higher prevalence of thyroid disease (5.79% vs. 4.19%), migraine (3.73% vs. 1.65%), arrhythmia (2.35% vs. 1.35%), hyperlipidemia (1.62% vs. 1.13%), and history of NSAID treatment (0.72% vs. 0.49%).

**Figure 2:**
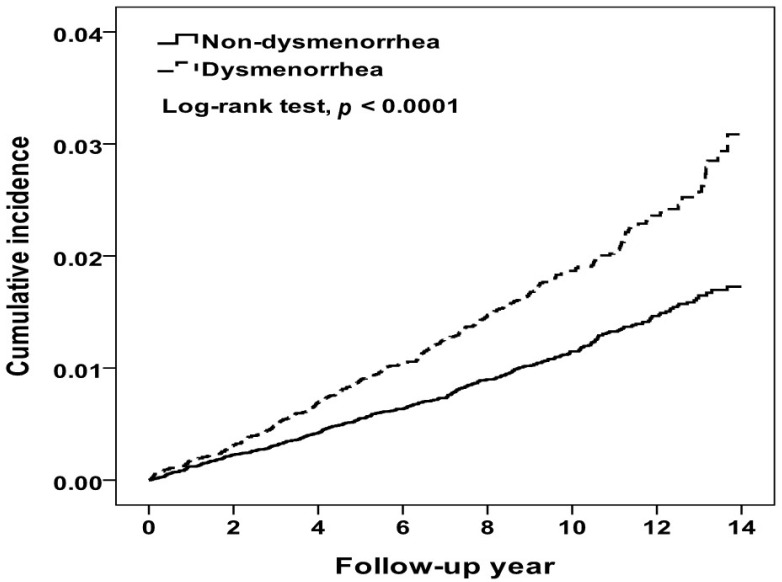
Kaplan-Meier method estimated cumulative incidence of ischemic heart disease between women with and without dysmenorrhea.

At the end of the 14-year follow up period, the cumulative incidence of IHD in the dysmenorrhea cohort was higher than that in the comparison cohort (3.09% vs. 1.73%, log-rank test *p* < 0.0001) (Figure 2). During a mean follow-up period of 9.67 years, 338 and 427 women were diagnosed with IHD in the dysmenorrhea and comparison cohorts, respectively (Table 2). Compared to women without dysmenorrhea, the dysmenorrhea cohort had a 1.64-fold higher incidence of IHD (1.93 vs. 1.18 per 10,000 person-years). The multivariable Cox model estimated an aHR for IHD of 1.60 (95% CI = 1.38–1.85) for the dysmenorrhea cohort relative to the comparison cohort, after controlling for covariates. The incidence increased with age in both cohorts, higher in the dysmenorrhea cohort with the age-specific aHR higher in 30–39 years old. Table 2 also shows that comorbidities were associated with increased risk of IHD in both cohorts, higher in the dysmenorrhea cohort. The aHR associated with comorbidity was significant for those with thyroid disease (aHR 1.79, 95% CI = 1.07–2.99). Furthermore, the NSAIDs- and hormones-users were seemed to have higher IR, but the events of IHD were rare.

Among women with dysmenorrhea, case-control analysis also showed that age was an important factor associated the IHD risk. Patients diagnosed with IHD were older than controls (37.8% vs. 6.50% aged 40–50 years-old), with an aOR of 11.3 (95% CI = 8.55–15.0) (Table 3). Comorbidities were also more prevalent among those diagnosed with IHD. Compared to those without IHD, patients with IHD had a higher incidence of hypertension (10.7% vs. 1.20%) and arrhythmia (11.2% vs. 2.18%) with aORs of 2.50 (95% CI = 1.64–3.81) and 3.30 (95% CI = 2.25–4.86), respectively.

## 4. Discussion

Studies have reported that PD shares some risk factors that are associated with developing IHD, such as stress, depression and lifestyle [8,35]. A study in China found that 178,205 healthy young women with menstrual abnormalities had an increased prevalence of hypertension [36]. To our knowledge, no population-based study has ever investigated whether women with PD are at an elevated risk of developing IHD due to similar risk factors. In this large, population-based 14-year observational study, the cumulative incidence of IHD increased steadily with the follow-up period in both cohorts, with the Kaplan-Meier curve increasing at a higher rate in the PD cohort than in the comparison cohort (3.09% vs. 1.73%, log-rank test *p* < 0.0001). Women with PD had a 60% greater hazard than women without PD to develop IHD by the end of follow-up period.

Our study showed that the incidence of IHD increased with age in both cohorts, but at a higher rate in the PD cohort than in the comparison. Compared to women aged 15–29 years old, the IHD incidence for those aged 40–50 years old in the PD cohort increased for 8.0 per 1000 person-years, compared to 6.82 per 1000 person-years in the non-dysmenorrhea cohort. In general, comorbidities associated with IHD may increase with age. It is likely that comorbidities are more prevalent in older women in both study cohorts. Data in the frequency matched cohorts showed that most comorbidities had no significant role in developing IHD, with the exception of thyroid disease. Nested case-control analysis within the PD cohort also showed that hypertension and arrhythmia played a significant role in developing IHD. It is possible that these comorbidities were more prevalent in older women with PD.

To date, there is no effective treatment to cure PD, although pharmacological and non-pharmacological therapy, and complementary and alternative medicine interventions have been used to care for women who suffer from dysmenorrheal pain and other symptoms [37,38]. Nonsteroidal anti-inflammatory drugs (NSAIDs) are prescribed most often, followed by hormonal contraceptives for women [19,37,38]. Previous studies have associated both types of medicines with an increased risk of developing IHD [39,40]. A systemic review reported that NSAIDs are associated with an increased risk of acute myocardial infarction with odds ratios ranging from 1.24 to 1.58 [39]. On the other hand, another systemic review on (non-)selective NSAIDs revealed no evidence for higher risk in bleeding or cardiovascular events in most studies [41]. We found that the adjusted HR of developing IHD in dysmenorrheal women was 1.60 (95% CI = 1.39–1.85) in non-NSAIDs users, whereas the adjusted HR was 1.76 (95% CI = 0.45–6.90) in NSAID users. These results indicate that NSAIDs played little role in the development of IHD in our study population. Hormonal contraceptive users were also not at an increased risk of IHD because few women used the medicine in both cohorts. This result was consistent with the previous study, which indicated none of victims took hormonal pills [26]. The effect of combined oral contraceptives is slow, patients are suggested to take more than 28 days for pain relief, but weight gain is of concern [42].It is interesting to note that the PD cohort to comparison cohort aHR of IHD for those with diabetes was 0.55 (95% CI = 0.27–1.10), which is insignificant. The nested case-control analysis in the PD cohort also showed that the aOR of IHD in those with diabetes was 0.72 (95% CI = 0.36–1.46). It is likely, based on this data, that diabetes is not as important as hypertension in the risk of developing IHD for women with PD. The nested case-control analysis also found that arrhythmia is a risk factor with an aOR greater than hypertension. Our findings are consistent with established knowledge that age, hypertension and arrhythmia are risk factors for IHD [43,44,45,46,47,48]. Atrial fibrillation has been associated with elevated mortality from heart failure with reduced ejection fraction in patients with IHD [47]. The American Heart Association (AHA) has raised the level of evidence (LOE) for atrial fibrillation complicating ACS from C to B, because treatment of AF is beneficial for IHD [48].

### Strengths and Limitations

By researching a large population, this study found a strong association between PD and IHD. The claims data were extracted randomly from the NIH of Taiwan with a stable population structure over time. We could thus apply a longitudinal design rather than have to use a cross-sectional approach to assess the innovation relationship between PD and IHD over time.

However, this study has limitations. First, the information on potential IHD-related risk factors such as tobacco use, body mass index, lipid profiles (the levels of LDL, HDL), exercise, and drinking was not available for evaluation [4,45]. Second, data of WBC count, hs-CRP and other biochemical markers of inflammation were also unavailable in the claims database. Third, not all the NSAIDs users were included in this study. Out of 18,455 women with dysmenorrhea, we found a history of NSAID use in only 132 of them, which was not consistent with dysmenorrhea treatment [19]. We hypothesize that many women with dysmenorrhea got the NSAIDs from drugstores, where they can be purchased over the counter in Taiwan. In spite of this, we believed that most women took NSAIDs intermittently, not chronically. NSAIDs confer the greatest risk for cardiovascular disease within the first month of use [39]. Accordingly, NSAIDs might be not a confounding factor for IHD in this study. Fourth, no specific test is available to be used to diagnose PD. We excluded women with SD based on identifying disorders that might associate with developing the dysmenorrhea. The procedure of pelvic examination is recommended for women with dysmenorrhea, but is not necessary for adolescents without sexual activity [19,27]. There might be diagnostic variability among physicians in their practices [49]. These biases might be overcome by using a large database, such as Taiwan’s insurance claims data [50]. Fifth, despite of the high prevalence of PD, some cases might be underdiagnosed or inadequately treated [22,26,51]. Information on these issues was unavailable in the claims data. Sixth, studies have reported women with high parity might be at an elevated risk of life time cardiovascular disease, including IHD and stroke, etc. [52,53,54]. It is unlikely we are able to observe this relationship in the 338 cases of IHD in young women with PD. Finally, the pathogenesis of the relationship between PD and IHD development could not be confirmed in our observational study.

## 5. Conclusions

This study revealed that women with PD were at an elevated risk of developing IHD, which increased with age. However, the risk of developing IHD for younger PD aged 15–29 years was not lower than the older PD women aged 40–50 years, based on the HR relative to the comparisons. It is important to note that 75% of women with PD were young. Better monitoring of women with PD of all ages is needed to reduce the IHD risk, particularly for those with comorbidities of thyroid disease, hypertension and arrythmia.

## Figures and Tables

**Table 1:** Characteristics compared between women with and without primary dysmenorrhea.

Variable	Non-Dysmenorrhea Cohort (*n* = 36,910)		Dysmenorrhea Cohort (*n* = 18,455)		*p* Value *
	*n*	%	*n*	%	
**Age group (years)**					0.99
15–29	27,666	75.0	13,833	75.0	
30–39	6634	18.0	3317	18.0	
40–50	2610	7.07	1305	7.07	
Mean age, years (SD)	25.7	(8.03)	25.6	(7.90)	0.27
**Baseline comorbidities**					
Diabetes	450	1.22	233	1.26	0.66
Hypertension	494	1.34	253	1.37	0.75
Hyperlipidemia	416	1.13	299	1.62	<0.0001
Arrhythmia	500	1.35	433	2.35	<0.0001
Thyroid disease	1545	4.19	1068	5.79	<0.0001
Migraine	610	1.65	689	3.73	<0.0001
**Medicine use**					
NSAID	180	0.49	132	0.72	0.0007
Female hormone use	32	0.09	16	0.09	0.99
Follow-up year, mean (SD)	9.77	(2.61)	9.47	(2.62)	<0.0001

* Chi-square test, and a *t*-test. SD, standard deviation. The median follow-up period was 9.83 years and 9.52 years for the dysmenorrhea cohort and comparison cohort, respectively.

**Table 2:** Incidence of ischemic heart disease and primary dysmenorrhea cohort to non-dysmenorrhea cohort hazard ratio by age, comorbidity, and medicine use.

Variables	Non-Dysmenorrhea Cohort			Dysmenorrhea Cohort			Adjusted HR ^†^		*p* for Interaction
	Event	Person Years	IR ^†^	Event	Person Years	IR ^†^	(95%CI)	*p*-Value	
**Overall**	427	360,608	1.18	338	174,747	1.93	1.60 (1.38–1.85)	<0.0001	
**Age group (years)**									0.429
15–29	125	270,201	0.46	105	131,794	0.80	1.65 (1.27–2.15)	0.0002	
30–39	119	65,270	1.82	105	31,107	3.38	1.76 (1.35–2.29)	<0.0001	
40–50	183	25,138	7.28	128	11,847	10.80	1.39 (1.10–1.74)	0.005	
**Diabetes**									0.0002
No	389	356,486	1.09	328	172,607	1.90	1.71 (1.47–1.98)	<0.0001	
Yes	38	4122	9.22	10	2140	4.67	0.55 (0.27–1.10)	0.091	
**Hypertension**									0.224
No	369	356,126	1.04	302	172,580	1.75	1.66 (1.43–1.94)	<0.0001	
Yes	58	4482	12.94	36	2167	16.61	1.19 (0.78–1.81)	0.435	
**Hyperlipidemia**									0.083
No	395	357,092	1.11	314	172,190	1.82	1.64 (1.42–1.91)	<0.0001	
Yes	32	3516	9.10	24	2557	9.39	1.06 (0.62–1.81)	0.844	
**Arrhythmia**									0.568
No	394	355,942	1.11	300	170,856	1.76	1.63 (1.40–1.90)	<0.0001	
Yes	33	4666	7.07	38	3891	9.77	1.38 (0.86–2.21)	0.180	
**Thyroid disease**									0.461
No	400	345,999	1.16	303	164,974	1.84	1.59 (1.36–1.84)	<0.0001	
Yes	27	14,609	1.85	35	9774	3.58	1.79 (1.07–2.99)	0.026	
**Migraine**									0.114
No	407	355,242	1.15	315	168,561	1.87	1.64 (1.41–1.90)	<0.0001	
Yes	20	5366	3.73	23	6186	3.72	1.14 (0.62–2.12)	0.669	
**NSAID**									0.728
No	423	358,803	1.18	332	173,423	1.91	1.60 (1.39–1.85)	<0.0001	
Yes	4	1805	2.22	6	1324	4.53	1.76 (0.45–6.90)	0.419	
**Hormones**									0.848
No	426	360,275	1.18	337	17,459	1.93	1.60 (1.38–1.85)	<0.0001	
Yes	1	333	3.00	1	158	6.33	2.24 (0.13–38.6)	0.580	

Abbreviation: IR, incidence rates, per 1000 person-years; HR, hazard ratio; CI, confidence interval. Adjusted HR ^†^ represented adjusted hazard ratio: mutually adjusted for dysmenorrhea, age, all comorbidities, and NSAID use in Cox proportional hazard regression.

**Table 3:** Nested case-control analysis for factors associated with ischemic heart disease in the primary dysmenorrheal cohort.

**Variable**	**Ischemic Heart Disease**							
	**Yes (*n* = 338)**		**No (*n* = 18,117)**		**Odds Ratio (95% Confidence Interval)**			
	***n***	**%**	***n***	**%**	**Crude**	***p*** **-Value**	**Adjusted**	***p*** **-Value**
**Age group (years)**								
15–29	105	31.1	13,728	75.8	1.00		1.00	
30–40	105	31.1	3212	17.7	4.27 (3.25–5.62)	<0.0001	3.95 (3.00–5.21)	<0.0001
40–50	128	37.8	1177	6.50	14.2 (1.09–18.5)	<0.0001	11.3 (8.55–15.0)	<0.0001
**Baseline comorbidities (yes vs. no)**								
Diabetes	10	2.96	223	1.23	2.45 (1.29–4.65)	0.006	0.72 (0.36–1.46)	0.370
Hypertension	36	10.7	217	1.20	8.93 (6.79–14.2)	<0.0001	2.50 (1.64–3.81)	<0.0001
Hyperlipidemia	24	7.10	275	1.52	4.96 (3.22–7.64)	<0.0001	1.30 (0.79–2.15)	0.302
Arrhythmia	38	11.2	395	2.18	5.69 (4.00–8.08)	<0.0001	3.30 (2.25–4.86)	<0.0001
Thyroid disease	35	10.4	1033	5.70	1.91 (1.34–2.73)	0.0004	1.17 (0.80–1.71)	0.407
Migraine	23	6.80	666	3.68	1.91 (1.24–2.94)	0.003	1.26 (0.81–1.98)	0.308
Medicine use								
NSAID	6	1.78	126	0.70	2.58 (1.13–5.90)	0.025	1.95 (0.81–4.71)	0.136
Hormones	1	0.30	15	0.08	3.59 (0.47–27.2)	0.216	NA	

## Data Availability

This study used the insurance claims data of Taiwan. Data are available on request from the insurance authority.
